# Supplementary material for: Cutaneous Melanoma Systematic Diagnostic Workflows and Integrated Reflectance Confocal Microscopy Assessed with a Retrospective, Comparative Longitudinal (2009–2018) Study
Source: Cancers (Basel). 2022 Feb 7;14(3):838. doi: 10.3390/cancers14030838 (PMC8834176; doi:10.3390/cancers14030838)
Supplement: Supplementary file 1 [file cancers-14-00838-s001.zip › cancers-1530145-supplementary-final.pdf]

cancers MDPI

Supplementary

Cutaneous Melanoma Systematic Diagnostic Workflows and Integrated Reflectance Confocal Microscopy Assessed with a Retrospective, Comparative Longitudinal (2009–2018) Study

Giovanni Pellacani, Francesca Farnetani, Johanna Chester, Shaniko Kaleci, Silvana Ciardo, Sara Bassoli, Alice Casari, Caterina Longo, Marco Manfredini, Anna Maria Cesinaro, Francesca Giusti, Antonio Iacuzio and Mario Migaldi

Table S1. Population statistics for the Province of Modena (2009-2018) according to age groups.

Age groups	2009	2010	2011	2012	2013	2014	2015	2016	2017	2018
0–49	417,234	419,524	421,606	421,639	419,412	412,521	407,999	403,284	399,421	395,518
50–70	172,102	173,944	176,311	178,767	181,289	183,828	187,438	190,915	195,417	197,228
>70	98,950	101,112	102,997	104,758	105,716	106,412	107,677	108,282	108,111	110,457
Total	688,286	694,580	700,914	705,164	706,417	702,761	703,114	702,481	702,949	703,203

Table S2. Crude prevalence of melanoma over the study period, based on population statistics.

Age groups	2009	2010	2011	2012	2013	2014	2015	2016	2017	2018
0–49 anni	1.5	1.9	1.4	1.6	1.2	2.1	2.1	3.1	3.2	4.1
50–70 anni	4.8	5.1	3.7	3.7	4.5	6.9	7.2	8.9	8.6	13.0
>70 anni	5.9	5.7	5.7	6.4	5.3	9.0	8.1	12.5	13.7	13.4
Total	2.9	3.2	2.6	2.9	2.7	4.4	4.4	6.1	6.3	8.1

Table S3. Standardized prevalence of melanoma over the study period, based on estimates from 2009.

Age groups	2009	2010	2011	2012	2013	2014	2015	2016	2017	2018
0–49	1.5	1.9	1.4	1.6	1.2	2.1	2.0	3.0	3.0	3.9
50–70	2.0	5.1	3.8	3.9	4.8	7.3	7.8	9.8	9.8	14.9
>70	1.4	5.9	6.0	6.8	5.7	9.7	8.8	13.6	15.0	15.0
Total	2.9	3.3	2.7	2.9	2.7	4.5	4.4	6.2	6.4	8.2

Table S4. All lesions. Melanoma. Nevi and respective NNEs according to location of excision at the Department of Dermatology (DP) or other health institutions in the province (NDP).

Year	Melanoma		Nevi		Total			Thin		Thick	
	N	%	N	%	N	%	NNE	N	%	N	%
DP											
2009	133	7.1	1744	92.9	1877	14.5	14	96	72.2	37	27.8
2010	99	6.3	1475	93.7	1574	12.1	16	79	79.8	20	20.2
2011	108	8.0	1244	92.0	1352	10.4	13	69	63.9	39	36.1
2012	92	9.9	836	90.1	928	7.2	10	58	63.0	34	37.0
2013	92	11.5	711	88.5	803	6.2	9	66	71.7	26	28.3
2014	163	17.5	768	82.5	931	7.2	6	126	77.3	37	22.7
2015	176	14.5	1041	85.5	1217	9.4	7	152	86.4	24	13.6
2016	220	16.3	1132	83.7	1352	10.4	6	179	81.4	41	18.6
2017	235	16.5	1187	83.5	1422	11	6	208	88.5	27	11.5
2018	310	20.4	1211	79.6	1521	11.7	5	282	91.0	28	9.0

Total	1628	12.5	11349	87.5	12977	100	8	1315	80.8	313	19.2
NDP											
2009	70	3.3	2066	96.7	2136	7.7	31	58	82.9	12	17.1
2010	126	4.6	2600	95.4	2726	9.8	22	113	89.7	13	10.3
2011	75	2.7	2706	97.3	2781	10	37	45	60.0	30	40.0
2012	109	3.9	2681	96.1	2790	10	26	85	78.0	24	22.0
2013	97	3.7	2522	96.3	2619	9.4	27	74	76.3	23	23.7
2014	146	5.4	2576	94.6	2722	9.8	19	129	88.4	17	11.6
2015	130	4.9	2518	95.1	2648	9.5	20	115	88.5	15	11.5
2016	209	7.2	2706	92.8	2915	10.5	14	180	86.1	29	13.9
2017	207	6.2	3120	93.8	3327	11.9	16	179	86.5	28	13.5
2018	257	8.1	2934	91.9	3191	11.5	12	231	89.9	26	10.1
Total	1426	5.1	26,429	94.9	27,855	100	20	1209	84.8	217	15.2
